# 2D Hexagonal Boron Nitride (h-BN) and 1D Boron Nitride Nanotubes (BNNTs): Distinct Effects at the Cellular Level in Fish Cell Lines

**DOI:** 10.3390/jox15040097

**Published:** 2025-06-24

**Authors:** Mona Connolly, Emmanuel Flahaut, José María Navas

**Affiliations:** 1 Instituto Nacional de Investigación y Tecnología Agraria y Alimentaria (INIA), Consejo Superior de Investigaciones Científicas (CSIC), Carretera de la Coruña Km 7,5, E-28040 Madrid, Spain; connolly.mona@inia.csic.es; 2 Centre National de la Recherche Scientifique (CNRS), Toulouse INP, Centre Interuniversitaire de Recherche et d’Ingénierie des MATériaux (CIRIMAT), Université de Toulouse, 118 Route de Narbonne, 31062 Toulouse Cedex 9, France; emmanuel.flahaut@univ-tlse3.fr

**Keywords:** 2D materials, multi-walled nanotubes, nanosheets, boron nitride, h-BN, BNNT, in vitro, rainbow trout (*Oncorhynchus mykiss*), cytotoxicity

## Abstract

Hexagonal boron nitride (h-BN) and boron nitride nanotubes (BNNTs) are emerging advanced nanomaterials with analogous structures to graphene and carbon nanotubes, respectively. However, little is known about what effect replacing carbon atoms with boron and nitrogen will have on the materials’ safety profile. This study’s aim was to first identify if multi-walled nanotubes of BN could produce a hazard profile similar to that evidenced already for multi-walled carbon nanotubes (MWCNTs) and secondly if the material when present in a sheet-like structure increases or decreases the hazard profile. Fish are aquatic organisms sensitive to boron compounds; however, the potential hazard following exposure to BN and especially when present in such nanostructures has not yet been investigated. An in vitro testing platform consisting of multiple cell lines of the rainbow trout, *Oncorhynchus mykiss* (RTH-149, RTG-2, RTL-W1 and RTgill-W1), was used in a first-hazard screening approach for cytotoxicity and to gain information on material–cellular interaction. Clear differences were evidenced in material uptake, leading to plasma membrane disruption accompanied with a loss in metabolic activity for BNNTs at lower exposure concentrations compared to h-BN. As in the case of carbon nanotubes, close attention must be given to potential interferences with assays based on optical readouts.

## 1. Introduction

The classification of nanomaterials based on dimension has been used to collectively refer to layered nanomaterials with a sheet-like structure as two-dimensional (2D) materials. Perhaps the most well-known materials within this class are the graphene related 2D materials. However, there are a number of other emerging 2D materials with extraordinary properties yet to be explored. Such properties are conferred upon these nanomaterials not only by their geometry but also due to their crystal structure form and unique chemistry. For instance, the 2D material hexagonal boron nitride (h-BN) shows an sp2 hybridization in the B and N atoms that form hexagonal rings in a layered structure similar to that of graphene. In addition, BN can be arranged in one-dimensional (1D) BN nanotubes (BNNTs) that have gained a lot of attention as structural analogues to carbon nanotubes (CNTs). Despite the structural similarity between these nanostructures of BN to that of graphene and CNTs, BN materials show unique physico-chemical properties, including electrical insulation, optical transparency, radiation resistance and superior thermal stability [1,2]. For example, BNNTs can act as both a good electrical insulator (due to the wide bandgap ~6 eV) while still conducting heat, unlike CNTs, which have both high electrical and thermal conductivity. This, together with the material’s high mechanical strength and resistance, is a rare and a useful combination, which makes BN very appropriate for use in the fabrication of nanocomposites with enhanced properties (e.g., reinforced polymers, ceramics and alloys or highly thermoconductive and electrically insulating polymeric composites [3]. Such nanocomposites are being investigated for their use in space and aerospace technologies [4], in electronics requiring heat dissipation and for novel emerging applications in many sectors [5]. In fact, the market for BNNTs for composite materials is expected to undergo rapid growth driven by industry demand for high-performance materials and commercialization, with a projected compound annual growth rate (CAGR) of 14.52% and a market value of 3.1 billion USD by 2032 [6]. Meanwhile, h-BN use as a 2D semiconductor material is expected to be harnessed across the electronics industry. Additionally, its application in anti-corrosive and high-temperature protective paints and coatings is seen as one of the fastest growing applications for h-BN [7].

These distinct physico-chemical properties may also have implications when comparing the potential hazardous effects for risk assessment. There is already quite a comprehensive amount of information from studies on 2D graphene sheets and 1D CNTs, which could be helpful in determining the extent to which material geometry may be contributing to any effects evidenced. For instance, there has already been some evidence to suggest a similar mechanism of acute pulmonary inflammatory toxicity for high-aspect-ratio thin and long BNNTs (0.6–1.6 µm long; 13–23 nm diameter) and MWCNTs (3.86 µm long; 49 nm diameter) in mice with similar pro-inflammatory cytokine profiles and pyroptosis due to NLRP3 inflammasome activation [8,9]. Chronic inflammation and an adaptive immunity response have also been reported as being associated with a long-term biopersistance following long-term exposure to similar BNNT materials (≥5 µm long; ≤8 nm in diameter) [10]. For 2D h-BN a similar response was not initiated, and no overt pathogenicity was evidenced in mice following oropharyngeal aspiration, which suggests a different hazard profile for these layered sheet-like structures that may more closely fit with responses seen following similar 2D graphene nanosheet exposure [11,12]. In the field of ecotoxicity, much less is known. This is worrying as the use of BN is increasing as well as the likelihood of these substances being released to the environment in the process of manufacturing, transport, use, recycling or final disposal. Considering that the final fate of environmental pollutants are aquatic environments, it is essential to gather data about the deleterious effects of BN for aquatic organisms.

An effective concentration causing a 50% reduction in growth (EC50) of 1.27 mg/L toward the freshwater algae, *Chlorella vulgaris*, for h-BN was reported, suggesting that this substance could show a higher toxicity than graphene (reduced, oxidized and multilayer) to this first-trophic-level organism [13,14]. Authors point to shading and aggregation contributing to such effects on algal growth for h-BN [15]. However, no significant effect on the mobility of the water flea, *Daphnia magna,* was evidenced when exfoliated h-BN was tested (up to 100 mg/L) [16]. The only published study on the effects of BNNTs on aquatic species is that of Evariste and colleagues on *Xenopus laevis* tadpoles, showing no obvious acute effects (up to 10 mg/L) [17]. Otherwise, the effects of 2D h-BN nanosheets or 1D BNNTs on higher-tier aquatic organisms such as fish have not been studied. Fish serve as important biomonitoring tools for the overall health of aquatic ecosystems, and some of the endpoints that can be assessed include changes in development, growth, reproduction and behavior [18]. Furthermore, investigations using microscopy techniques (e.g., atomic force or transmission electron microscopy) can be used to detail interactions of these nanoscale materials with aquatic organisms at a single cell level to characterize the specific interactions of these nanomaterials at the nanoscale [19].

At the cellular level, similar membrane damaging effects as those reported for graphene sheets have been evidenced in algae [13], bacteria [20] and lung epithelial cells H460 [21] as well as the human liver carcinoma cell line, HEP G2 [22], following exposure to h-BN nanosheets. Early studies on cytotoxic effects of BNNTs by Horvath and colleagues [23], as well as more recent publications [24,25], also point to potential cytotoxicity for these nanotube materials; however, other BNNTs are reported as being biocompatible [26,27]. As seen for CNTs, cellular interaction and uptake is likely dependent on the nanotube length, rigidity and flexibility (bending stiffness) and/or biopersistance of the nanotube itself [28]. Of course, BNNT uptake will also be influenced by the cell line model used with differences in cytotoxicity in macrophages vs. kidney cell lines reported by [23] and also for primary mouse epithelial vs. human alveolar epithelial cells by [29]. This discrepancy, together with the strong interferences in the assays evidenced for these materials—with one of the most widely used cytotoxicity assay tetrazolium dyes, MTT [30]—makes comparisons or conclusions on hazards at the cellular level challenging.

Taking into account all of the above, the main aim of this study was to screen for the potential hazardous effects of h-BN and BNNTs using in vitro approaches based on fish cell lines and to compare how the material structural form can influence their safety profiles. In vitro systems provide a highly valuable initial approach for hazard assessment. Their use not only leads to significant resource savings but also aligns with the principles of the 3Rs. In this work the fibroblast-like fish gonad cell line, RTG 2, as well as the epithelial-like cell lines from the gill, liver and hepatoma of rainbow trout, *Oncorhynchus mykiss* (RTgill-W1, RTL-W1 and RTH-149, respectively), were selected for use. Three cytotoxicity assays performed on the same set of cells that assess alterations in metabolic activity, plasma membrane integrity and lysosomal functioning were applied. This approach is the one used in the OECD TG 249 fish cell line acute toxicity test [31], which utilizes the RTgill-W1 cell line. In addition, the potential involvement of oxidative stress processes was assessed by measuring any increases in the cellular levels of reactive oxygen species (ROS) while transmission electron microscopy was employed in order to directly visualize cellular interaction, uptake and processing of the materials. An important emphasis has been put on the assessment of potential interferences by these materials with the assay systems applied due to the high adsorption capacity, inherent photoluminescence and potential light-shading properties of these advanced materials. Material interference with assay systems can lead to misleading results and incorrect interpretations. Yet despite early publications highlighting this [32,33,34], it is often neglected. This has been recently highlighted by Andraos and colleagues [35] and even efforts have been made to develop decision trees and approaches for interference testing to tackle these issues [36]. In this study, we have developed an interference test protocol for the assays being applied to show how such assessments can be and need to be incorporated in parallel with such cytotoxicity assays.

## 2. Materials and Methods

### 2.1. Hexagonal Boron Nitride (h-BN) and BNNTs

Hexagonal boron nitride (h-BN) was supplied in powder form by BeDimensional, Genoa, Italy. These h-BN flakes are few-layered and were prepared by liquid-phase exfoliation (LPE) of pristine bulk h-BN using N-methyl-2-pirrolidone (NMP) and a high-pressure wet-jet-milling (WJM) technique. The h-BN flakes have a lateral size of ~130 nm (TEM analysis), thickness of 2.54 nm (AFM analysis), <1% wt residual solvent and surface area of 19 m^2^/g (BET analysis) (according to the information provided from the producer). The tapped density of the exfoliated h-BN is 0.166 g/cm^3^.

The boron nitride nanotubes (BNNTs) used in this study were synthesized and provided by BNNT, LLC, USA (BNNT SP10RX). These refined, highly crystalline and purified nanotubes were produced by the catalyst-free high temperature/high pressure (HTP) method, also known as the pressurized vapor condenser (PVC) method. This particular material (SP10RX) has been refined using a high-temperature steam purification process (U.S. Patent US11629054B2) [37] to remove non-nanotube BN species (e.g., elemental boron) and only contains boron nitride (99%). The BNNTs are few-walled, with manufacturer-reported sizes of 5 nm in width and >200 μm in length, with a measured specific surface area (BET, TriStar II 3020, Micromeritics, Norcross, GA, USA) of 405 m^2^/g. The band gap is 5.7 eV and the materials are flexible with an extremely low defect density. This SP10RX BNNT material was provided by the manufacturer in a refined puffball form.

### 2.2. Preparation and Characterisation of Exposure Suspensions

The h-BN-exfoliated powder was dispersed in miliQ water (2 mg/mL) and sonicated for 30 min in an ultrasonic water bath at 37 kHz (total energy delivered: 36,826 J) (Fisherbrand S-series; Thermo Fisher Scientific, Waltham, MA, USA). Overheating of the sample was prevented by cold-water replacement in the water bath after 15 min. This stock dispersion was used to prepare the exposure concentrations in culture medium according to the cell line being tested. For the in vitro studies, the BNNT puffball sample needed to be suspended in a compatible vehicle. A total of 15 mg of dry BNNTs was weighed and placed in a glass vial. A total of 15 mL of absolute ethanol was added progressively with 1 min of bath sonication in between two additions. The sample was then processed using a small-diameter (3 mm) ultrasound probe for 3 min, 1 s On: 1 s Off, with an amplitude of 30%. The suspension was washed with milli-Q water on a filtration system with a polypropylene membrane (0.45 µm). The product was collected from the membrane and the milli-Q water was added to reach a final concentration of 2 mg/mL. The suspension was finally placed in an ultrasonic bath (Bioblock Scientific model 75042, Vibracell, Bioblock Scientific, Illkirch, France (500 W, 20 kHz)) and sonicated for 1 h using two cycles of 30 min each. Transmission electron microscopy was used to characterize the dispersed BNNT and h-BN materials, using a TEM JEOL JEM 1400 ORIUS (JEOL Ltd., Tokyo, Japan) operating at an electron acceleration voltage of 120 kV.

Dynamic light scattering (DLS) analysis using a Zetasizer Nano-ZS apparatus (Malvern Instruments Ltd., Malvern, UK) was also used to characterize the size–frequency distributions of the dispersions (both stock and highest exposure concentrations (200 µg/mL)). Three measurements were taken of each sample and any changes in the size distribution over time in the culture medium were assessed according to the measurements taken after 24 h.

### 2.3. Cell Culture and Exposure

All the cell lines used were from rainbow trout. The RTL-W1 liver cell line was a generous gift from Dr. Bols and Dr. Lee [38] and they are non-transformed epithelial-like cells isolated from a normal liver of the rainbow trout (*Oncorhynchus mykiss*) [39,40]. The RTgill-W1 gill epithelial cell line [41], the RTH-149 liver hepatoma cell line [42] and the fibroblast-like RTG-2 gonadal cell line [43] were all sourced from the American Type Culture Collection (ATTC) (Manassas, VA, USA). The cells were cultured in 75 cm^2^ cellstar cell culture flasks (Greiner Bio-One GmbH, Frickenhausen, Germany) incubated at 20 °C, and in the case of the RTH-149 and RTG-2 cell lines, they were maintained under 5% CO_2_ atmosphere. Leibovitz’s L-15 culture medium (Gibco; Thermo Fisher Scientific) was used to culture the RTL-W1 and RTgill-W1 cell lines, while the RTH-149 and RTG-2 cell lines were cultured in MEM Eagle with Earle’s balanced salt solution (PAN-BiotecH, Aidenbach, Germany). All the media were supplemented with 10% fetal bovine serum (FBS) (Sigma Aldrich; Merck group, Darmstadt, Germany) and 1% penicillin/streptomycin (P/S) solution (Lonza; Thermo Fisher Scientific). The cells were split regularly using 0.5% (*w*/*v*) trypsin and 0.01% (*w*/*v*) EDTA in phosphate-buffered saline (PBS).

The experiments were carried out on confluent cell monolayers obtained with a seeding density of 2.5 × 10^4^ cells/well (100 µL culture media) after 24 h of culture in 96-well plates (Greiner-Bio one, CellStar, Spain). Using 200 µg/mL as the highest exposure concentration, the cells were exposed to a 1:2 dilution concentration series of the h-BN and BNNT materials for 24 h. This exposure concentration expressed in µg/cm^2^ is equivalent to 62.5 µg/cm^2^, and when expressed in material surface area per cellular surface area (assuming complete delivery of dose), it is equivalent to a 0.11875 cm^2^ h-BN/cm^2^ cell and 1.1425 cm^2^ BNNT/cm^2^ cell. As a vehicle control culture medium with 10% *v/v,* miliQ water was used, and as a positive control for the ROS assay, copper(II) nitrate (100 µM) was used.

### 2.4. Interaction with Cells and Uptake

Transmission electron microscopy (TEM) was used to investigate the interaction of the h-BN and BNNT materials with the RTgill-W1 cells and their possible uptake. This cell line is representative of the other cells used in this study with an epithelial-like morphology and thus cellular-interaction studies were only performed using this representative cell line. The cells were seeded on poly-L-lysine-coated cover slips in 24-well plates (Greiner-Bio one, CellStar, Madrid, Spain) at a density of 1.5 × 10^5^ cells/well (equivalent seeding density per cm^2^ to 96 well format) and were exposed to 25 μg/mL (7.821 µg/cm^2^) equivalent to 0.1484 cm^2^ h-BN/cm^2^ or 1.42 cm^2^ BNNT/cm^2^ for 24 h. After exposure, the samples were prepared as described by [40], including the washing steps (Millonig phosphate buffer, pH 7.3), primary fixation (4% paraformaldehyde/2.5% glutaraldehyde), post fixation (1% osmium tetroxide), gradual dehydration steps (30–100% acetone), embedding (gradual infiltration with Spurr’s resin) and polymerization step (65 °C, 48 h). Ultrathin sections were stained in uranyl acetate and lead citrate and viewed in a JEOL 1010 JEM TEM (JEOL Ltd., Tokyo, Japan).

### 2.5. Cytotoxicity Assays

Cytotoxicity was assessed in each of the rainbow trout cell lines: RTgill-W1, RTL-W1, RTH-149 and RTG-2. The cells were exposed to a concentration range of 1.56–200 µg/mL (0.488–62.5 µg/cm^2^), and according to the distinct BET (Brunauer, Emmett and Teller) surface areas of the h-BN and BNNT materials, this was equivalent to a substance surface area per cell surface area dose of 0.0092–1.187 cm^2^/cm^2^ and 0.089–11.41 cm^2^/cm^2^ for the h-BN and BNNT, respectively. Three assays were used to assess any impairment of the cellular activities and to aid in understanding the mechanisms of cytotoxicity behind any effects seen. The AlamarBlue^TM^ (AB) (resazurin) dye (Invitrogen; Thermo Fisher Scientific), the 5-carboxyfluorescein diacetate-acetoxymethyl ester (CFDA-AM) probe (Invitrogen; Thermo Fisher Scientific) and the neutral red (NR) dye (amino-7-dimethylamino-2-methylphenazine hydrochloride) (Sigma Aldrich; Merck group) served to detect alterations in the cellular metabolic activity, plasma membrane and lysosomal membrane integrity, respectively. The use of these three indicator dyes together and simultaneously as a new approach methodology was first described by [44], modified and applied for nanomaterial testing by other authors [45] and more recently utilized in the OECD TG 249 RTgill-W1 fish cell line acute toxicity test [31].

After 24 h exposure, the medium containing the h-BN and BNNT was removed and the cells were washed twice with PBS. The cells were incubated with AB (1.25% *v*/*v*)/CFDA-AM (4 µM) working solutions prepared in phenol red-free culture medium under dark conditions for 30 min. Thereafter, the fluorescence at excitation (exc.)/emission (em.) wavelengths, 530 nm/595 nm and 493 nm/541 nm, respectively, were read using a Spark 20 M microplate reader (Tecan Genios, Tecan Group Ltd., Männedorf, Switzerland). Following the removal and washing of the AB/CFDA-AM solution, a NR dye solution (0.03 µg/mL) prepared in phenol red-free culture medium was applied to the cells for 1 h under dark conditions. After a washing step with PBS, the retained NR was extracted using acidified (1% glacial acetic acid) 50% ethanol/49% Milli-Q water solution. The fluorescence was then measured at 532 nm exc. and 680 nm em. For all the assays, fluorescence readings in blank wells (AB, CFDA-AM and NR dye only) were subtracted from those in the cell-treated wells and the resulting relative fluorescence units were presented as a % of the control untreated cell well fluorescence readings.

### 2.6. Reactive Oxygen Species (ROS) Generation

The 6-carboxy-2′7′dichlorodihydrofluorescein diacetate (DCFH-DA) probe (Merck group, Spain) was used to measure any increases in the levels of ROS in the RTgill-W1 cells following the h-BN and BNNT exposures. First, the cells were exposed to the h-BN and BNNT materials for 24 h. Thereafter, the exposed cells were washed twice with PBS and incubated under dark conditions with 100 μM of the DCFH-DA probe (30 min). Thereafter, the probe was removed and the cells were washed with PBS. The fluorescence readings (485 nm exc/535 nm em) were taken immediately (t0) and after 60 min. The percentage increase in fluorescence per well was calculated using the formula [(Ft60 min − Ft0)/Ft0 × 100], where Ft60 min and Ft0 are the fluorescence measured at time 60 min and 0 min, respectively. Increases in the ROS levels are expressed according to the % increase in fluorescence with respect to the control cells (100%).

### 2.7. Assay Interference Screening Studies

The experiments were performed to check for potential interferences with the assays due to autofluorescence, fluorescence quenching and/or interactions of the h-BN and BNNT materials with the indicator dye reagents (e.g., dye adsorption) that may contribute to false reporting. The described methodology can be used as a first-screening approach to provide information about the potential interference issues when performing the assays using cells.

Autofluorescence was measured by preparing the concentration ranges of the materials (1.56–200 µg/mL) in assay medium (phenol red and serum-free) and measuring any fluorescent signals at each of the assay excitation and emission fluorescence wavelengths (AB: 530 nm/595 nm, CFDA-AM: 493 nn/541 nm and NR: 530 nm/635 nm).

In a second experiment, to assess if the material could reduce (quench) the fluorescent signal from the respective assay reaction products/fluorophores, suspensions of resorufin (1 µM), 5-carboxyfluorescein (5-CF) (4 µM) and acidified NR (0.03 µg/mL) were prepared. These were dispensed into 96-well plates and the exposure concentration ranges of the h-BN and BNNT were introduced. Thereafter, the fluorescence at the assay wavelengths (as detailed above) was measured across the entire plate to compare the fluorophore-only values and those at increasing concentrations of the material.

In the third approach, to assess the adsorption properties, each of the assay’s reagents were prepared (resazurin (1.25% *v*/*v*), CFDA-AM (4 µM) and NR (0.03 µg/mL)) and incubated with the h-BN and BNNT material concentration ranges in micro-centrifuge tubes for 30 min in the case of resazurin and CFDA-AM and 1 h in the case of NR (in order to mimic the assay incubation times). Thereafter, the samples were centrifuged (10,000× *g*) for 30 min and the supernatant fluorescence was measured (resazurin: fluorescence 530 nm/595 nm, CFDA-AM: fluorescence 493 nm/540 nm and NR: absorbance 540 nm) and compared to the samples only with the assay reagents. Any obvious loss in signal/color from the supernatant was indicative of dye adsorption. In addition, a visual inspection of the material pellet was used to confirm any adsorption.

In all the interference experiments, any reduction or increase in the measured fluorescence/absorbance was expressed as a % of the background or fluorophore/reagent-only signal. These experiments were performed without the need for cells in the plates.

The ROS assay is a kinetic-based assay; therefore, one would assume that any background interference would be read at t0 and thus any further increases in the signals measured could only be due to increases in the ROS levels. However, the potential of these materials to autofluoresce, to oxidate the deacetylated DCFH-DA probe to the fluorophore dichlorofluorescein (DCF) or to quench/block its fluorescence was assessed. Fluorescence readings (485 nm exc/535 nm em) of increasing concentrations of the h-BN and BNNT materials alone were taken and compared to the medium-only values to check for autofluorescence. To observe potential oxidation or quenching, any decrease or increase in the fluorescence values in the cells that had taken up the probe was compared with the fluorescence measurements taken directly after the probe addition and when these cells were exposed to increasing concentrations of the h-BN and BNNT materials. The fluorescence readings were then taken at 485 nm exc./535 nm em. and compared to the values in the cells with the probe only.

### 2.8. Data Interpretation and Statistical Analysis

The cytotoxicity assays and ROS level results are expressed as the mean ± standard error of the mean (SEM) of at least three independent experiments, in which each treatment was applied in triplicate in each culture plate. The normality (Kolmogórov–Smirnov test/Shapiro–Wilk test) and the homogeneity of variance (Bartlett’s test) were checked prior to carrying out the statistical analysis. The normal and homoscedastic data were analyzed for significant differences in response by a one-way analysis of variance (ANOVA) followed by Dunnett’s post hoc test (treatment vs. control) in SigmaPlot^®^ 12.0 (Systat Software Inc., Chicago, IL, USA). This allowed for the identification of the lowest observed effect concentrations (LOECs) for each assay. The IC50 (concentration causing a 50% inhibition with respect to the controls) values were also calculated using non-linear regression curve fitting of log-transformed data and applying a hill slope in GraphPad Software. All the graphs were prepared using GraphPad Prism 10.0 (GraphPad Software, Inc., La Jolla, CA, USA).

## 3. Results

### 3.1. h-BN and BNNT Material Characterization

**Figure 1:**
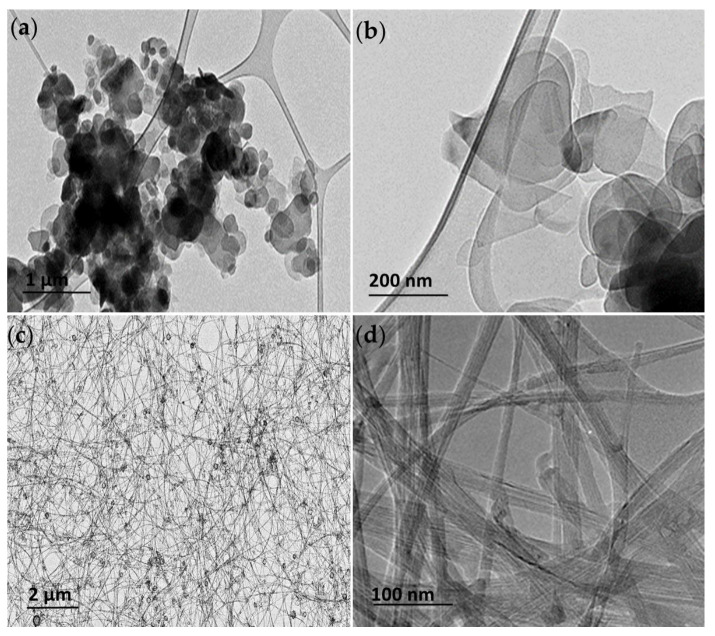
Transmission electron microscopy (TEM) images of the h-BN (**a**,**b**) and BNNT materials (**c**,**d**) at low and high magnification showing the distinct structure and characteristic shapes of the h-BN and BNNTs used in this study.

TEM was used to visualize the h-BN and BNNT and assess their shape and size. The hBN particles were found present in an aggregated state on the grids; however, individual hBN particles of flakes with round-shaped edges could be identified with 50% of the population with mean diameters of 100–400 nm (Figure 1a,b), consistent with the characterization of this material performed by other authors [8]. The BNNT puffball sample after dispersion in ethanol consisted of a tangled arrangement of long (>200 µm reported by manufacturer) flexible multi-walled BNNTs, among which there is also a small proportion of hBN empty-faceted onions (Figure 1c). Individual thin few-layered BNNTs with widths of 10–20 nm could be identified (Figure 1d).

Stock suspensions of the h-BN material were prepared in MiliQ water following water bath sonication to aid dispersion (36,826 Joules total delivered energy). The DLS analysis evidenced a particle diameter of 456 ± 65 nm for h-BN in this stock dispersion (Table 1). The hydrodynamic size distribution of the nanosheets was maintained when the material was diluted in the L-15 (RTL-W1 and RTgill-W1) and EMEM (RTG 2 and RTH-149) cell culture media used in the exposures. While a DLS analysis is not the most appropriate technique to characterize the size of non-spherical-shaped particles such as nanotubes, it was used here to monitor any changes in the distribution/agglomeration state when the BNNT material was prepared in culture medium. The size distributions were the same in the stock MiliQ and cell culture medium suspensions (graphical depictions of the size distribution can be found in the Supplementary Information). The BNNT suspensions were not subjected to sonication in order to preserve the structure and nanotube form as it has been shown to possibly change the tube length [46]. Thus, the TEM images presented are truly representative of the materials used in the exposures to the cells, and the medium does not have a dramatic effect on either of the materials’ size distribution.

### 3.2. Cellular Interaction and Uptake

**Figure 2:**
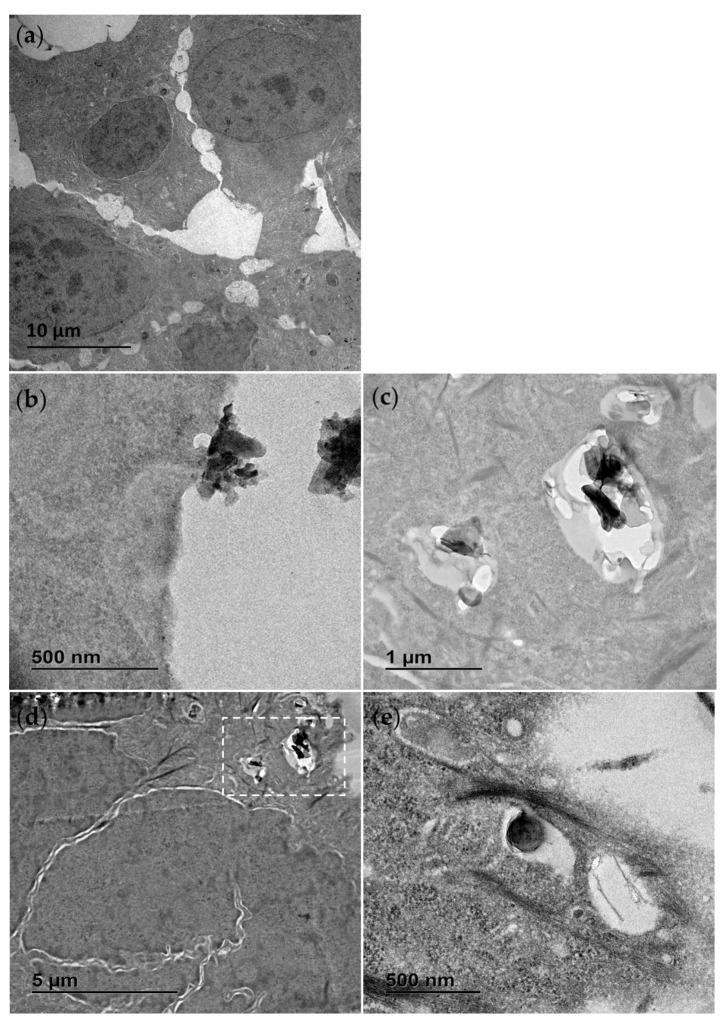
Transmission electron microscopy (TEM) images of RTgill-W1 cells untreated (**a**) and exposed for 24 h to 25 µg/mL h-BN showing close interaction with the plasma membrane (**b**) and uptake into the intracellular vesicles (**c**–**e**). (**c**) is the section of (**d**) marked with the white dotted line box.

TEM was also used to study the interaction of the materials with exposed cells. Following an exposure duration of 24 h to 25 µg/mL (middle of concentration range tested), the treatments were removed, and the cells were washed with PBS, fixed and prepared for analysis. In Figure 2 the contrasting appearance of the h-BN nanosheets and BNNTs, respectively, can be seen, as well as the distinct cellular interaction and processing compared to unexposed control RTgill-W1 cells (Figure 2a). In Figure 2b an agglomerate of h-BN sheets can be seen adhered to the cellular membrane with indications of the involvement of membrane invagination in the cell’s particle processing mechanism. The cellular uptake of these nanosheets and agglomerates is evidenced and their capture in intracellular vesicles confirmed (Figure 2d). This is the case for single h-BN sheets approx. 300 nm in diameter (Figure 2e) and bigger agglomerates up to 1 µm in size (Figure 2c).

**Figure 3:**
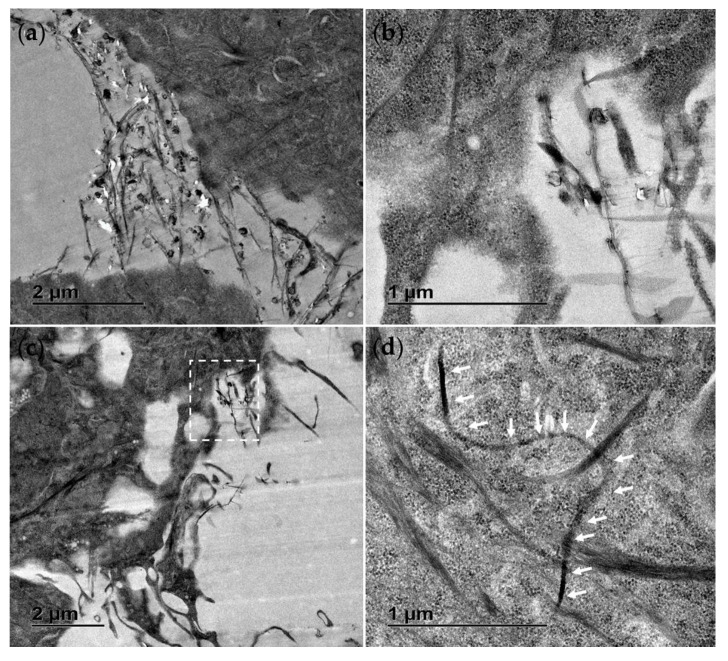
Transmission electron microscopy (TEM) images of RTgill-W1 cells exposed for 24 h to 25 µg/mL BNNT showing material direct entry across the plasma membrane (**a**) as well as widespread distribution free in the cytosol (**b**–**d**). (**b**) is the section of (**c**) marked with the white dotted line box. White arrows indicate the distinct flexible structure of the BNNT.

In contrast, the BNNT materials due to their flexible structure were found dispersed, tangled and interacting with the membrane with random orientations, sometimes even appearing to pierce through the cellular membrane (Figure 3b,c). There was no evidence of a cellular processing mechanism for these materials and instead they appeared to be found traversing the plasma membrane and in direct contact with cellular organelles (Figure 3d). Due to the thin fibrous nature of the BNNT tubes resembling cellular microtubules and filaments found within the cytosol of the control cells, they could only be distinguished by their distinct flexible geometry and when seen in direct interaction with the cellular plasma membrane.

### 3.3. Cytotoxicity Assessment

#### 3.3.1. Assay Interference Screening Studies

The assays used in this study to assess cytotoxicity rely on optical read-outs, specifically fluorescence readings. They use cell-permeable indicator dyes and probes, and according to the assay protocols, these are directly applied to cells, which have been exposed to the h-BN and BNNT materials. Therefore, it is likely that any material taken up or associated with cells or in the wells of the plate will come into contact with these dyes/probes. Such interactions may lead to assay interferences, and this has been checked following an in-house-developed interference protocol.

Inherent fluorescence signals from the material themselves that could contribute to the assay readout values were first assessed. Both the h-BN and BNNT materials showed a concentration-dependent increase in the fluorescent signal at the fluorescence wavelengths used in the AlamarBlue (AB) assay (Table 2). The h-BN materials had higher fluorescence values, reaching a 184% increase from the background control levels, and the BNNT materials reached a 60% increase from the background levels at the highest exposure concentration. At the wavelengths of fluorescence used in the CFDA-AM assay, only the BNNT showed a fluorescence increase at a similar level seen for the AB assay wavelengths. At the neutral red assay wavelengths, increases in the fluorescence were evidenced for both the h-BN and BNNT and caused a 150% and 50% increase in the fluorescence values, respectively.

In the second set of interference studies, the materials were incubated with the products of the assays, and the extent to which the inherent fluorescence of the materials, or indeed any quenching, might influence the readings was assessed. Interestingly, a concentration-dependent reduction in the resorufin (AlamarBlue assay product) fluorescence values was measured at the higher BNNT concentrations, resulting in only 72% of the product-alone control values at the highest concentration of 200 µg/mL. When incubated with 5-CF, the materials only caused slight deviations from the dye-only control values. A significant reduction in the acidified neutral red fluorescence was only evidenced at the higher concentrations of BNNTs.

Finally, in a set of experiments to assess potential adsorption, the materials were incubated with the assay reagents, a centrifugation step was used to precipitate all the material and the concentration of the reagent remaining in suspension (not adsorbed) was measured. Any decrease in the fluorescence/absorbance signal compared to the assay reagent-only control samples was used to detect binding/adsorption and potential assay interference. The measurements revealed no decrease in the resazurin concentration when incubated with either of the materials (Table 2). However, a strong binding for the CFDA-AM reagent to BNNTs was evident (reducing the signal by up to 58%). In the case of the neutral red dye, reductions of 10% and 11% were seen for both materials at the highest concentrations. It is also worth noting that binding of the neutral red dye to the nanomaterials was visually evidenced as the material pellets following centrifugation exhibited a red color. This is likely to lead to false high fluorescence values in the NR assay due to the contribution from the dye adsorbed to the materials taken up/adhered to the well that can not be differentiated from any accumulation or loss of dye in intact lysosomes.

#### 3.3.2. AlamarBlue (AB) Assay Results (Considering Interferences)

**Figure 4:**
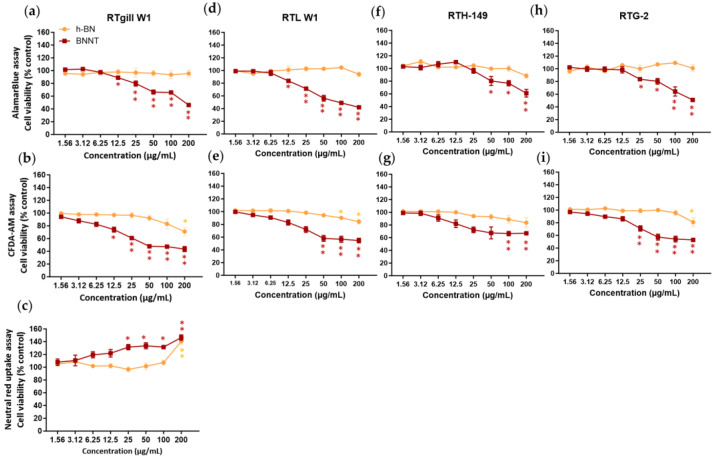
Cytotoxicity assessment comparison of h-BN nanosheets and BNNTs using rainbow trout cell lines and the AlamarBlue (**a**,**d**,**f**,**h**), CFDA-AM (**b**,**e**,**g**,**i**) and neutral red (**c**) assays. Exposure concentrations are nominal and mass-based. Equivalent surface area-based concentrations tested are 0.49–62.5 µg/cm^2^. * *p* < 0.005; ** *p* < 0.001.

This assay based on the conversion of the resazurin dye to fluorescent resorufin product was applied to the cells after 24 h exposure to measure the levels of metabolic activity. h-BN did not cause any significant reductions in metabolic activity from the control cells at concentrations up to 200 µg/mL in any of the rainbow trout cell lines (Figure 4). While the interference screening studies showed that the h-BN material had a high inherent autofluorescence, the use of a washing step prior to the application of the resazurin dye likely reduces the interference potential so that a real lack of cellular toxicity was observed.

In contrast to the lack of cytotoxicity seen for h-BN, we have evidenced a significant (*p* < 0.05) dose-dependent decrease in metabolic activity following BNNT exposure. The levels of viability were reduced to between 42 and 61% of the control levels depending on the cells tested when exposed to the highest concentration of BNNTs (200 µg/mL). Significant cell viability decreases, indicating a LOEC value, were already observed at concentrations of 12.5 µg/mL for RTgill-W1 and RTL-W1, at 50 µg/mL for RTH-149 and at 25 µg/mL for RTG-2 cells. The BNNT materials exhibited both potential autofluorescence and fluorescence quenching properties. However, as significant reductions in viability were already evidenced at concentrations at which little interferences were detected, the observed reduction in cell viability can be relied upon. Using a curve fitting, IC50 values of 150 ± 14 µg/mL, 201 ± 92 µg/mL, 382 ± 98 µg/mL and 255 ± 117 µg/mL could be calculated for the RTgill-W1, RTL-W1, RTH-149 and RTG-2 cells, respectively, following 24 h BNNT exposure.

#### 3.3.3. CFDA-AM Assay Results (Considering Interferences)

The CFDA-AM assay measures the plasma membrane integrity of cells, based on the entry of this cell-permeable probe into cells and its conversion by esterase activity to a highly fluorescent product, 5-CF, that is retained in cells with intact plasma membranes. In cells exposed to h-BN materials, decreases in the cell viability of a maximum of approximately 20% that lead to significant (*p* < 0.05) differences with respect to controls in the 5-CF signal were measured at the highest concentration (LOEC, 200 µg/mL) for RTgill-W1 and from 100 µg/mL (LOEC) in RTL-W1 cells. However, the BNNT-exposed cells showed a clear concentration-dependent effect with significant (*p* < 0.05) viability decreases starting at concentrations of 12.5 µg/mL (LOEC, RTgill-W1 cells), 50 µg/mL (RTL-W1), 100 µg/mL (RTH-149) and 25 µg/mL (RTG-2) (Figure 4). Taking into consideration any potential interferences and the data presented above (Section 3.3), the BNNT materials did not appear to be able to cause any quenching in the 5-CF assay product signal; however, they showed a strong binding affinity for CFDA-AM, which could lead to a lack of availability of CFDA-AM to cells causing false-positive results. The BNNT material also showed autofluorescence; therefore, any material attached to the surface of the well may contribute to the signal and thus increase the fluorescence values. This may explain the flattening of the line seen at the high exposure concentrations instead of a further reduction in viability.

A curve fitting could not be performed on this data; however, a LOEC value of 12.5 µg/mL for the BNNT and 200 µg/mL for the h-BN was calculated from the statistical analysis (RTgill-W1 cells).

#### 3.3.4. Neutral Red (NR) Assay Results (Considering Interferences)

This assay was used to measure any reduction in viability associated with lysosomal membrane disruption. Neutral red dye readily enters cells and accumulates in intact lysosomes of healthy cells. Any reductions in levels of dye would suggest lysosomal disruption. The results showed no reduction in the levels of dye measured in the cells treated with the h-BN or BNNT (RTgill-W1 cells) (Figure 4c). Instead, in the case of h-BN, higher levels were measured at the highest exposure concentration (141% viability) and this same increase was more dramatically evidenced in the case of BNNT-exposed cells and in a concentration-dependent manner with already higher values measured at 25 µg/mL (132% viability).

This is likely explained by the ability of these materials to autofluoresce and also the high adsorption capacity of the materials for the NR dye evidenced in the interference screening studies. Any material taken up by cells or left on the wells will adsorb excess NR dye and this will contribute to higher values in the readout in wells exposed to higher concentrations. Due to this obvious interference of the h-BN and BNNT materials with this assay, it was not performed when testing the cytotoxicity in the other cell lines.

### 3.4. Reactive Oxygen Species (ROS) Level Assessment

To assess if exposure to these materials caused an increase in ROS levels that may suggest oxidative stress involved in cellular processing, the DCFH-DA probe was used. As this assay relies also on a fluorescent readout, any potential autofluorescence or quenching by the materials was screened for prior to performing the assay. The results revealed that the BNNTs have a high inherent autofluorescence at the assay wavelength (485 nm exc/535 em.) compared to the h-BN test materials (Table 3). These BNNT materials also show a fluorescent quenching ability causing up to a 60% reduction in the DCF product fluorescence. The h-BN materials show little autofluorescence; however, they did increase the DCF fluorescent signal by 10–20%.

It must be noted however that the ROS assay protocol used [47] is a kinetic-based assay and involves exposing the cells first, removing the treatment, washing with PBS and adding the probe and measuring any increases over time in fluorescence from intracellular probe deacetylation caused with ROS. Therefore, the reading at t0 will act as a background (including any interference from material left in the well of the plate or internalized by cells) and any increases in fluorescence measured thereafter could only be due to the deacetylation reaction.

**Figure 5:**
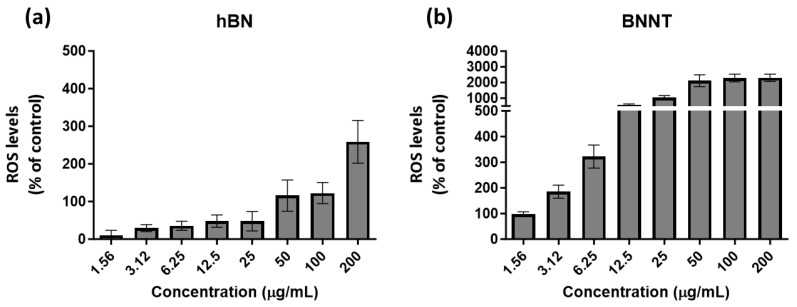
Levels of ROS compared to control-untreated cells as measured using the DCFH-DA probe following 24 h exposure to the h-BN (**a**) and BNNTs (**b**) in RTgill-W1 cells.

The % of control levels measured for h-BN material-exposed cells started below the basal cellular control ROS levels (100%). However, following exposures to concentrations of 200 µg/mL, the h-BN levels increased to levels above the control and over 2-fold higher (Figure 5a). In contrast, the cells exposed to low concentrations of BNNTs had the same basal ROS levels as the control (100%), but this increased already following 3.12 µg/mL exposure and this effect was heightened with increased exposure concentrations reaching 10- to 20-fold higher levels than the control levels (Figure 5b).

## 4. Discussion

The results from comparing the hazard profile of the different BN nanomaterials (2D h-BN nanosheets and 1D BNNTs) using rainbow trout fish cell lines show that the BNNTs cause a significant reduction in cellular viability at lower exposure concentrations than hBN and thus present a higher potential hazard profile. This result is consistent in all the fish cell lines tested (RTgill-W1, RTL-W1, RTH-149 and RTG-2). The effects on the plasma membrane recorded using the CFDA-AM assay point to a unique interaction of these long tube-like structures with the cellular membrane involved in their toxicity mechanism. This assay also recorded plasma membrane disruption for the h-BN nanosheets but to a much lesser extent. The distinct interaction of the tested BNNT materials with the plasma membrane was confirmed by direct visualization of the cells following exposure by transmission electron microscopy. From the TEM images, it could not be concluded that active cellular uptake processes were involved. Instead, individual nanotubes appear to be penetrating/piercing the plasma membrane and present diffusely in the cytoplasm. This corroborates the results from the CFDA-AM plasma membrane disruption assay and also the associated loss in metabolic activity recorded according to the AB assay.

However, this cellular-interaction mechanism is in contrast to active uptake processes, such as phagocytosis or endocytosis, seen for other structurally similar 1D materials, such as CNT clusters, which can result in a frustrated phagocytosis leading to cell death [48]. The difference is likely related to the distinct size, as the BNNTs tested here are very long (>200 μm) and their geometry/flexibility may not favor active uptake, in contrast with the shorter CNTs (<20 µm) [49]. Instead, membrane adsorption and piercing appear to be facilitating their uptake. Such a mechanism has also been described for individual CNTs and is referred to as passive diffusion [50,51]. This highlights that the specific dimensions of the nanotubes and aggregation state will likely dictate the interaction, processing and effect. In addition, these very long and flexible BNNTs may not fit the fiber pathogenicity paradigm (FPP) put forward for long and rigid nanotubes and instead present a distinct hazard profile. For example, the LOEC of 12.5 µg/mL is lower than that observed for the much shorter MWCNT-NM-400 (length 0.8−1.5 μm) (LOEC 128 µg/mL) when tested using the same fish cells (RTG-2) and cytotoxicity assay (CFDA-AM assay) [52]. Other authors have also shown such a size-dependent effect on cytotoxic potential when comparing short and long BNNTs and confirmed a greater effect for the longer tubes [24]. This has also been evidenced in the case of CNTs when comparing the cytotoxicity of short and longer nanotubes [53].

In contrast, for the tested h-BN nanosheet nanostructures, their size and geometry (~130 nm, lamellar disc-shaped) favored active internalization through an endocytic pathway involving membrane invagination and the processing of the nanosheets in vacuoles in RTgill-W1 cells. Material uptake was not associated with any loss in cellular metabolic activity and only very subtle effects on plasma membrane integrity were recorded at high exposure concentrations (200 µg/mL) in all the fish cells. This appears to be distinct for the h-BN nanosheets with a rounded border used in the present study, as nanosheets of h-BN with specific sharp-cornered edges have shown increased cytotoxicity associated with the ability of their exposed polar edges to penetrate lipid bilayers and to form cross-membrane water channels [21]. Such sharp-edged h-BN materials caused significant lysosomal membrane permeabilization, apoptosis and cell death in lung epithelial H460 cells exposed to 20 µg/mL hBN nanosheets [21]. In fact, increases in membrane fluidity (measured using a fluorescence probe, TMA-DPH) and transmembrane efflux pump activity inhibition have even been evidenced at concentrations as low as 0.5 μg/mL and 0.2 μg/mL, respectively, in the human hepatocellular carcinoma cell line Hep G2 [22]. Significant deleterious effects on human alveolar epithelial A549 cell and corneal epithelial hTCEpi cell viability have also been measured at 40 µg/mL and 4 µg/mL, respectively (Calcein AM assay) [29]. Also h-BN nanosheets caused strong concentration-dependent hemolytic activity (not evidenced in the case of an analogous graphene-based material) [54]. A greater cytotoxic effect towards human umbilical vein endothelial HUVEC cells was also evidenced compared to the analogous graphene-based material (clonogenic assay) [55]. Also when comparing the cytotoxicity of a series of 2D materials, h-BN nanosheets (along with reduced graphene nanosheets) were the only materials to cause any reduction in viability of primary mouse tracheal epithelial cells (mTEC) (albeit at high concentrations of 200 µg/mL) [29]. This discrepancy between the lack of cytotoxic effects for the h-BN used in this present study and the effects in studies mentioned could be explained by the differences in material structural features and in particular the rounded vs. sharp edges.

While the results from this study point to a lower hazard potential for the h-BN nanosheets tested compared to the BNNT materials, the results from interference studies show that there is a need for comprehensive evaluation beyond the traditional viability assays and to use assays that do not rely on optical readouts. The interference studies developed for the assays used in this study were incorporated in parallel and afforded a screening of possible interference potential from the different materials in each of the assays. According to the results from autofluorescence screening, it becomes evident that a potential contribution from material-inherent fluorescence is possible in all three assays (with the exception of h-BN at the CFDA-AM assay fluorescence). The incorporation of the washing steps following cellular exposure will reduce the level of interference when performing the assay. Furthermore, due to the assay principles, any increased signal would signify an increase in viability, and thus, if reductions are measured, this is real and certainly not overestimated. This is the case for effects measured for BNNTs using the AB assay.

In this study both the h-BN and BNNT materials were found to interact directly (adsorb) with the NR dye, and at the time of extraction resulted in high fluorescent values, suggesting greater than 100% viability of material-exposed cells. These experiments could not be relied upon and thus this assay was deemed unreliable for use when testing these materials. Such associations are caused by interactions between the BN surface hydroxyl group and NR functional groups and in fact this has been described for BN and other nanomaterials with high adsorption properties for NR, such as graphene oxide [56]. This points to the need for new alternative/adapted assays for cytotoxicity assessment of such advanced materials as well as very thorough interference studies. One example of an alternative assay that does not rely on optical measurements is the non-colorimetric and non-fluorescent colony-forming efficiency assay that has been optimized and standardized for nanomaterial testing as an early screening method for basal cytotoxicity [57]. In fact, a reduction in the number of cells following hBN exposure was reported by means of the colony-forming efficiency assay that was not detected using the traditional tetrazolium salt-based CCK8 and MTT cell viability assays [55]. Another alternative is the use of electrical impedance-based assays with the advantage that they can be applied in real time [58]. In a novel approach, an AFM-based cardiomyocyte assay has been developed and used to test the cytotoxicity of BNNTs by measuring the beating pattern of Cor.At murine iPSC-derived cardiomyocytes and categorizing the cells into groups based on their visual health status [24]. This assay confirmed a concentration-dependent cytotoxic effect of BNNTs already at concentrations of 5 µg/mL.

A clear induction of ROS was evidenced in the cells after exposure to both BN materials. Using analogous-sized materials, there was a more pronounced increase in intracellular ROS levels for h-BN vs. graphene in human umbilical vein endothelial HUVEC cells, suggesting also a boron material-specific contribution to the effect [55]. As well as producing greater cytotoxic effects, we have evidenced that the BNNTs caused a much greater increase in cellular ROS and at lower exposure concentrations compared to h-BN. The ROS levels were already significantly increased from the control levels at 3.12 µg/mL and a 10-fold increase was seen at higher concentrations (≥25 µg/mL). Explanations for this include the presence of unsaturated boron atoms present at the material edges, which are chemically active and react readily with other reactive atoms such as oxygen [59]. This effect could also be caused by the materials’ nanotube geometry, as ROS-associated cytotoxic effects have also been commonly reported following CNT exposure [53]. Just as the BNNTs caused a strong increase in the ROS levels in the RTgill-W1 cells, a MWCNT (length 1->10 μm-BayTubes^®^ C 150 P) also caused significant increases in the RTL-W1 cells, reaching 4.7 times higher than the control levels following 24 h exposure to 50 µg/mL [60]. However, the extent of involvement of ROS in cytotoxicity likely depends on the different lengths, diameter and rigidity of these nanotubes as evidenced when different-sized MWCNTs were compared [61].

Another key factor to take into consideration in this hazard assessment is that the distinct uptake mechanisms seen for the different BN nanostructures is likely to affect intracellular concentration (delivered dose). A detailed study on the intracellular dose will be needed to derive specific IC50 values based on the actual delivered dose for the respective materials. Many authors have reported differences in delivered doses for different nanotubes [62] that depend on many factors, but often the actual delivered dose does not reach > 50% of the nominal dose after 24 h exposure [62,63,64]. Therefore, if this is the case for the BNNTs, the hazard and the IC50 of 150 µg/mL reported in this study will be even underestimated using the nominal concentration in this case.

In addition, what happens to these materials once in the cells for a prolonged time remains unanswered, and depending on the cellular processing mechanisms, it may lead to different chronic long-term effects. For the h-BN, it seems that after a long-term repeated exposure regimen (twice per week for 4 weeks) (1 μg/mL), there were no effects on the human HaCaT skin cells or the bronchial epithelial lung BEAS-2B cells’ viability (LDH assay) [65]. For BNNTs there have been no long-term studies. This is a critical knowledge gap as already the malignant transformation of human lung epithelial cells following CNT long-term exposure has been reported [66]. Furthermore, any intracellular degradation will likely result in cells being exposed to transformation products or the boron and nitrogen source precursors (e.g., boric acid, B(OH)_3_). Early publications on this issue highlighted that h-BN degradation will very much depend on the particular precursors used to produce the h-BN materials [67]. While hBN is likely to have a higher resistance to oxidation from enzymatic degradation than typical inorganic 2D materials [68], it can be degraded by human myeloperoxidase (hMPO)), forming soluble boric acid [69]. Boric acid has well known negative effects on the male and female reproductive systems, and it is classified as toxic to reproduction under “Category 1B” with the hazard statement of “H360FD” in the CLP Regulation (European Regulation on Classification, Labelling and Packaging of substances and mixtures) [70]. Thus, it will be pertinent to study the degradation potential of these materials (both in the environment, in organisms and at the cellular level). This has been highlighted in a recent review [71], which has revealed a serious data gap in our knowledge on the environmental and biological degradability of 2D materials, which limits the extent of our understanding on the potential risks of these emerging advanced materials.

## 5. Conclusions

In this study, we have demonstrated how the 1D and 2D BN material-specific form (geometry) and elemental composition can influence its hazard potential at the cellular level using fish cells as a model system. The increased hazard from the BNNTs compared to h-BN nanosheets fits with the aspect ratio-dependent increased hazard paradigm also evidenced when comparing 1D CNTs and 2D graphene effects both in mammalian and fish cells [72,73] and in recent in vivo studies in mice comparing the pulmonary effects of BNNT and h-BN [10]. In the present work, h-BN materials with rounded edges were tested, which caused little disruption to the plasma membrane; however, an increased cytotoxicity will likely result for materials with rough edges as evidenced for graphene 2D nanosheets. In the case of 1D nanotubes, large and flexible BNNTs were assessed, and they were internalized into cells by passive diffusion, albeit resulting in significant plasma membrane damage. Shorter BNNTs will likely be taken up by cells in a distinct manner and result in different hazard profiles as seen in the case of CNTs. The contribution of h-BN material-specific properties, such as unsaturated B atom reactivity that will result in distinct oxidative stress processes, as well as the contribution of known hazardous degradation products such as boric acid, to effects must be considered. Finally, the results from the developed interference screening show that this is a crucial aspect to be included in these kinds of approaches. Actually, strong interferences leading to increased or decreased readouts were detected and they must be considered in the interpretation of the final results. Therefore, one must conclude that for advanced material testing, in vitro cell culture-based investigations can be improved with interference-free assays and by measuring the delivered doses in order to meet the need for more accurate dose–response data for hazard assessments. Once such areas have been addressed, this will foster the inclusion and increase the weight of such in vitro approaches using cell models in hazard assessments of the future. Such investigations at the cellular level provide key information on potential events involved in the processing of such materials that will be pertinent to identify in order to meet future safe- and sustainable-by-design needs.

## Figures and Tables

**Table 1:** Dynamic light scattering (DLS) characterization of h-BN and BNNT aqueous stock dispersions and test dispersions prepared in the different culture media used in exposures.

Time (h)	Dispersion Medium	Concentration (µg/mL)	Z-ave ^a^ (nm)	PDI ^b^	Mean Diameter (nm) Peak 1 ^c^	Mean Diameter (nm) Peak 2 ^c^
h-BN						
	Stock Mili-Q	2000	394 ± 12	0.263	456 ± 65	
24 h	L-15	200	486 ± 89	0.226	591 ± 112	
24 h	EMEM	200	460 ± 9	0.258	550 ± 38	
BNNT						
	Stock Mili-Q	2000	450 ± 20	0.568	1195 ± 187	243 ± 59
24 h	L-15	200	463 ± 11	0.715	1280 ± 111	262 ± 31
24 h	EMEM	200	468 ± 16	0.506	1314 ± 73	236 ± 23

^a^ Zeta Average, ^b^ the Polydispersity index and ^c^ peaks in the frequency size distribution according to the intensity measurements.

**Table 2:** Interference screening study results summary.

Concentration (µg/mL)	1.56	3.12	6.25	12.5	25	50	100	200
Autofluorescence (% compared to control; control: 100%) AlamarBlue Assay								
h-BN	96	97	100	107	117	144	199	284
BNNT	100	101	103	107	112	122	134	160
CFDA-AM assay								
h-BN	95	95	95	94	95	95	97	101
BNNT	94	95	95	101	106	122	137	167
Neutral red uptake assay								
h-BN	100	108	100	100	108	133	167	250
BNNT	108	108	108	125	125	125	125	150
Fluorescence quenching (% compared to control; control: 100%) AlamarBlue Assay								
h-BN	100	100	100	102	103	103	98	101
BNNT	96	98	98	97	96	92	84	72
CFDA-AM assay								
h-BN	99	101	101	100	99	99	91	93
BNNT	107	106	104	104	104	102	104	107
Neutral red uptake assay								
h-BN	100	102	102	102	102	103	97	101
BNNT	100	102	103	104	100	95	90	82
Adsorption interference (% compared to control; control: 100%) AlamarBlue Assay								
h-BN	87	88	90	94	92	88	89	99
BNNT	99	100	104	99	100	99	98	98
CFDA-AM assay								
h-BN	84	90	90	100	88	90	91	92
BNNT	89	83	81	80	74	64	53	42
Neutral red uptake assay								
h-BN	98	100	100	99	100	97	95	90
BNNT	104	102	101	101	99	99	94	89

**Table 3:** Interference screening study results for the DCFH-DA assay and h-BN and BNNT materials.

Concentration (µg/mL)	1.56	3.12	6.25	12.5	25	50	100	200
DCFH-DA assay Autofluorescence (% compared to control; control: 100%)								
h-BN	98	100	100	98	100	100	103	107
BNNT	105	102	110	106	106	111	136	162
Fluorescence quenching								
h-BN	106	102	105	110	112	115	116	120
BNNT	103	102	99	97	84	62	51	49

## Data Availability

The data presented in this study are available upon request from the corresponding author and will be openly available once published at https://digital.csic.es (accessed on 23 June 2025).

## Supplementary Materials

The following supporting information can be downloaded at https://www.mdpi.com/article/10.3390/jox15040097/s1, Supplementary Figure S1. DLS characterization of h-BN and BNNTs) in aqueous stock dispersions (Stock Mili-Q) and culture media test dispersions.

## Abbreviations

The following abbreviations are used in this manuscript:

hBN	Hexagonal boron nitride
BNNT	Boron nitride nanotube
1D	One dimensional
2D	Two dimensional
DCFH-DA	Dichloro-dihydro-fluorescein diacetate
LDH	Lactate dehydrogenase
CNT	Carbon nanotube
ROS	Reactive oxygen species
MWCNT	Multi-walled carbon nanotubes
NR	Neutral red
CFDA-AM	5-carboxyfluorescein diacetate acetoxymethyl ester
AB	AlamarBlue
AFM	Atomic force microscopy
TEM	Transmission electron microscopy
PBS	Phosphate-buffered saline
5-CF	5-carboxyfluorescein
DCF	2′,7′-dichlorofluorescin-diacetate
LOEC	Lowest observed effect concentration
OECD	Organisation for Economic Co-operation and Development
