# Supplementary material for: 2D Hexagonal Boron Nitride (h-BN) and 1D Boron Nitride Nanotubes (BNNTs): Distinct Effects at the Cellular Level in Fish Cell Lines
Source: J Xenobiot. 2025 Jun 24;15(4):97. doi: 10.3390/jox15040097 (PMC12286044; doi:10.3390/jox15040097)
Supplement: Supplementary file 1 [file jox-15-00097-s001.zip › jox-3609395-supplementary.pdf]

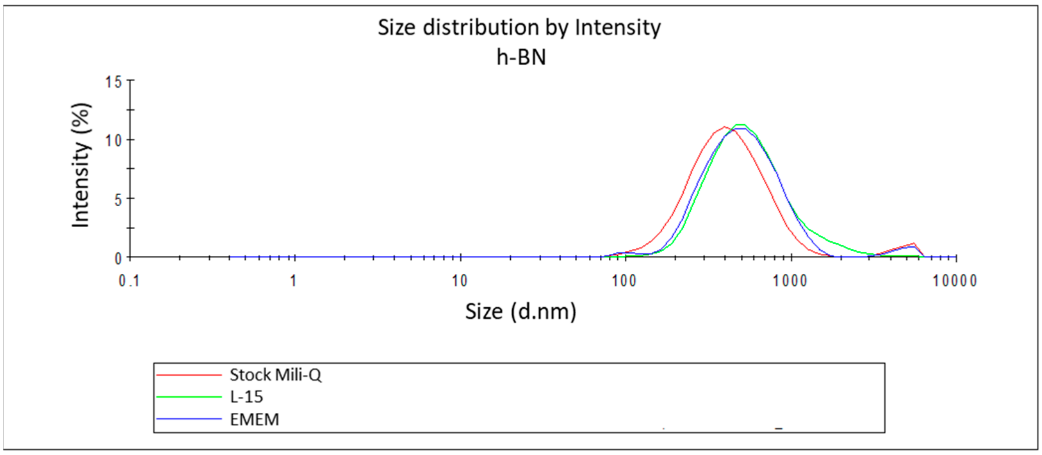

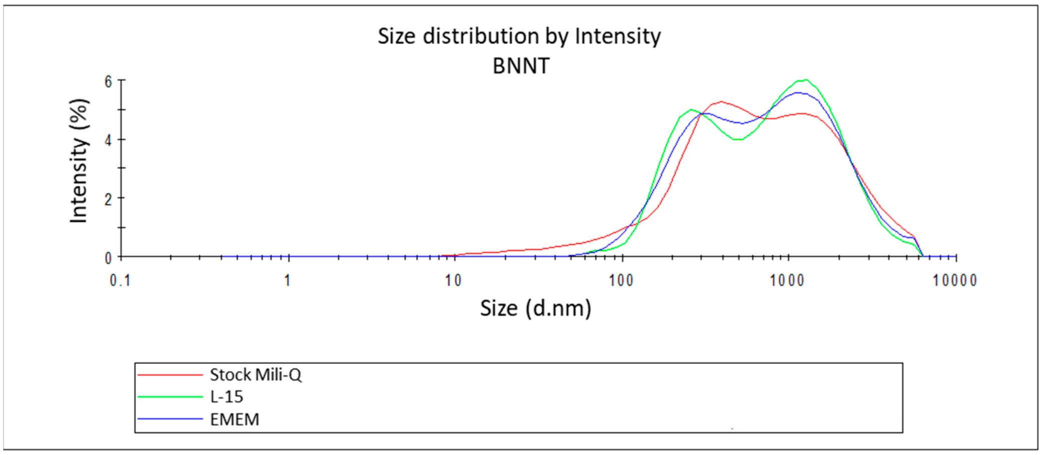

Supplementary Figure S1. DLS characterization of h-BN (upper) and BNNT (lower) aqueous stock dispersions (Stock Mili-Q) and test dispersions prepared in the different culture media used in exposures (L-15 (Lebowitz 15 medium) and EMEM (Eagle's minimum essential medium) and measured after 24h. The size distribution curves are presented by Intensity measurement and the size is reported in diameter (d.nm).
